# Carpal Tunnel Syndrome Is Associated with Increased Risk of Fibromyalgia: A Retrospective Cohort Study

**DOI:** 10.3390/life16071059

**Published:** 2026-06-25

**Authors:** Yu-Jung Su, Yun-Chen Liang, Yen-Po Chen, Wan-Yun Hsu, Hui-Chin Chang, Yu-Chao Tseng, Hsi-Chen Wei, Chun-Ming Chen, Shuo-Yan Gau

**Affiliations:** 1 Orthopedics Department, Chi-Mei Medical Center, Tainan 71004, Taiwan; 2 School of Medicine, Chung Shan Medical University, Taichung 40201, Taiwan; 3 Evidence-Based Medicine Center, Chung Shan Medical University Hospital, Taichung 40201, Taiwan; 4 Library, Chung Shan Medical University Hospital, Taichung 40201, Taiwan; 5 Division of Allergy, Immunology, and Rheumatology, Department of Internal Medicine, Ditmanson Medical Foundation Chia-Yi Christian Hospital, Chiayi 600566, Taiwan; 6 Department of Medical Research, Ditmanson Medical Foundation Chia-Yi Christian Hospital, Chiayi 600566, Taiwan; 7 Department of Biomedical Sciences, Institute of Molecular Biology, National Chung Cheng University, Chiayi 621301, Taiwan; 8 Institute of Allergology, Charité-Universitätsmedizin Berlin, Corporate Member of Freie Universität Berlin and Humboldt-Universität zu Berlin, 12203 Berlin, Germany; 9 Department and Graduate Institute of Business Administration, National Taiwan University, Taipei 10617, Taiwan; 10 Department of Medical Education, Ditmanson Medical Foundation Chia-Yi Christian Hospital, Chiayi 600566, Taiwan

**Keywords:** carpal tunnel syndrome, fibromyalgia, real-world study

## Abstract

**Background:** Carpal tunnel syndrome (CTS) is a common peripheral nerve entrapment disorder with multifactorial etiologies, while fibromyalgia is a chronic centralized pain condition characterized by widespread pain and central sensitization. Although shared mechanisms such as neurogenic inflammation and altered pain processing have been proposed, longitudinal evidence evaluating whether CTS predisposes to subsequent fibromyalgia remains limited. **Methods:** We conducted a retrospective cohort study using the TriNetX Global Collaborative Network. Adults aged ≥ 18 years with ≥2 clinical encounters between 2018 and 2023 were included. Patients with CTS formed the exposure cohort, while individuals without CTS undergoing routine health examinations served as controls. Those with prior fibromyalgia, malignancy, or death before index were excluded. One-to-one propensity score matching was performed to balance demographics, body mass index, psychiatric conditions, socioeconomic factors, healthcare utilization, and comorbidities including mood, anxiety, stress-related, and sleep disorders. The primary outcome was incident fibromyalgia. Sensitivity analyses included alternative matching strategies, extended washout periods, stricter exposure definitions, and active comparator analyses using osteoarthritis. Stratified analyses by age and sex were conducted. Associations were estimated using hazard ratios with 95% confidence intervals. **Results:** After matching, 217,208 patients were included in each cohort. CTS was associated with a significantly increased risk of fibromyalgia (HR 2.709, 95% CI 2.521–2.911). Consistent findings were observed across sensitivity analyses. Compared with osteoarthritis, CTS remained associated with higher fibromyalgia risk (HR 1.331, 95% CI 1.254–1.411). Stratified analyses demonstrated consistent associations across age groups (18–64 years: HR 2.820, 95% CI 2.595–3.065; ≥65 years: HR 2.717, 95% CI 2.337–3.159) and sexes (male: HR 3.018, 95% CI 2.482–3.672; female: HR 2.655, 95% CI 2.457–2.869). **Conclusions:** CTS was associated with coded fibromyalgia diagnosis in this large real-world cohort, and this association was observed across multiple sensitivity and stratified analyses. These findings should be interpreted as evidence of an epidemiologic association rather than a causal relationship. CTS may serve as a clinical marker for patients who warrant attention to broader pain-related symptoms, while future studies are needed to clarify temporality and underlying mechanisms.

## 1. Introduction

Carpal tunnel syndrome (CTS) is one of the most common peripheral nerve compression syndromes worldwide, resulting from compression of the median nerve at the wrist [1]. Patients typically present with nocturnal paresthesia and pain in the early stages, which may progress to irreversible sensory loss and thenar muscle atrophy in advanced cases. Epidemiologically, CTS affects approximately 1% to 5% of the general population, with a significantly higher prevalence in females than in males. The peak incidence falls within the age ranges of 50–54 and 75–84 years old [2]. It is often caused by a combination of factors. Essentially, any mechanism that raises pressure inside the carpal tunnel serves as an inciting factor. Established risk factors include diabetes, obesity, hypothyroidism, pregnancy, and high-intensity wrist activity; occasionally, CTS may also serve as an early warning sign for rare systemic diseases such as amyloidosis [1,2].

Fibromyalgia is a chronic, centralized pain syndrome primarily characterized by abnormal pain signal processing within the central nervous system and neurotransmitter imbalance, and it is strongly associated with reduced quality of life and a high prevalence of depression [3,4,5,6]. The condition most commonly presents with diffuse musculoskeletal pain, frequently accompanied by profound fatigue, disrupted sleep patterns, and impairments in cognitive function, often described clinically as “fibro fog” [5]. Fibromyalgia affects approximately 2% of the global population, with a higher prevalence in females than in males [7]. The peak age of onset typically falls between 50 and 60 years [8]. The exact cause of fibromyalgia is multifactorial, involving a complex interplay of various factors. Importantly, accumulating evidence from the literature suggests that chronic inflammatory diseases are associated with an increased risk of developing fibromyalgia, possibly through persistent immune activation and altered pain-processing pathways [9,10]. Additional contributing factors include obesity, certain infections, and chronic low-grade inflammatory responses [3,4,11].

Previous studies have mainly examined the prevalence of CTS among patients with established fibromyalgia [11,12], whereas longitudinal evidence evaluating the occurrence of fibromyalgia following CTS remains limited. The two conditions may present with similar pain-related symptoms and are often influenced by comparable risk factors [3,4,13]. Therefore, further population-based evidence is needed to clarify their temporal association. This retrospective cohort study aimed to evaluate whether individuals with CTS had a higher incidence of subsequently coded fibromyalgia diagnoses compared with individuals without CTS. The study was designed to assess an epidemiologic association rather than to establish causality or confirm a mechanistic pathway.

## 2. Materials and Methods

A retrospective cohort framework was employed for this analysis, drawing on data from the TriNetX Global Collaborative Network, a global electronic health record database widely used in pharmacoepidemiology and outcomes research [14,15,16]. This resource represents a large-scale, internationally federated system that aggregates de-identified electronic medical records contributed by multiple healthcare providers, with ongoing updates to ensure data recency. The subset of data used in the present study was obtained from the Global Collaborative Network, which includes participating institutions across regions such as the Americas, Europe, and Asia. This study adhered to internationally accepted ethical standards, specifically those outlined in the Declaration of Helsinki. The study protocol was reviewed and approved by the Institutional Review Board of Chi Mei Medical Center (approval number 11502-E02). Because the dataset consisted entirely of anonymized patient information, the requirement for informed consent was waived by the Institutional Review Board.

Eligible subjects were identified using standardized diagnostic and administrative coding systems (details provided in Table S1). Adult individuals (older than 18 years) were categorized into the exposure cohort if they met the criteria for CTS, defined as having at least two clinical visit records during the period from 1 January 2018 to 31 December 2023. Detailed information regarding disease severity was not available within the TriNetX platform. Therefore, CTS severity could not be incorporated into cohort definitions or subsequent analyses. A comparison cohort was constructed from individuals without CTS who had undergone routine health evaluations during the same period, identified using ICD-10-CM code Z00.0. Exclusion criteria were applied to remove individuals younger than 18 years, those with any prior diagnosis of malignancy, patients who became deceased before cohort entry, and those with a documented diagnosis of fibromyalgia prior to or on the index date. The primary outcome of interest was newly identified fibromyalgia, defined according to ICD-10-CM code M79.7.

To address potential baseline imbalances and reduce confounding effects, a 1:1 propensity score matching strategy was implemented. Matching variables encompassed demographic characteristics (including age, sex, and race), body mass index, psychiatric conditions related to substance use, psychosocial and socioeconomic factors, and patterns of healthcare utilization across both inpatient and outpatient settings. Additional comorbid conditions, such as mood disorders, anxiety-related disorders, stress-associated conditions, and sleep disorders, were also incorporated into the matching model. To validate the findings, supplementary analyses were performed, including alternative matching approaches and the application of washout periods designed to minimize reverse causation. Stratified analyses were further conducted based on age and sex categories. To evaluate the robustness of CTS identification, we also performed sensitivity analyses applying stricter claim-based exposure definitions. These included patients with a coded CTS diagnosis plus median nerve release surgery and patients with a coded CTS diagnosis plus a prescription for nonsteroidal anti-inflammatory drugs (NSAIDs). Direct neurophysiological confirmation, including nerve conduction studies or electromyography, was not available on the TriNetX platform. Furthermore, an active-comparator analysis was performed using patients with osteoarthritis. Osteoarthritis was selected because it is a chronic musculoskeletal condition commonly associated with persistent pain, functional impairment, and frequent healthcare utilization, thereby providing a clinically relevant comparator for partially addressing surveillance bias and differences in healthcare-seeking behavior.

Statistical procedures were conducted within the TriNetX analytical environment. The relationship between CTS and the subsequent risk of fibromyalgia was quantified using hazard ratios (HRs) with 95% confidence intervals (CIs). Post-matching balance between cohorts was assessed using standardized mean differences (SMDs), with a threshold of less than 0.1 indicating adequate balance.

## 3. Results

### 3.1. Baseline Characteristics

**Figure 1:**
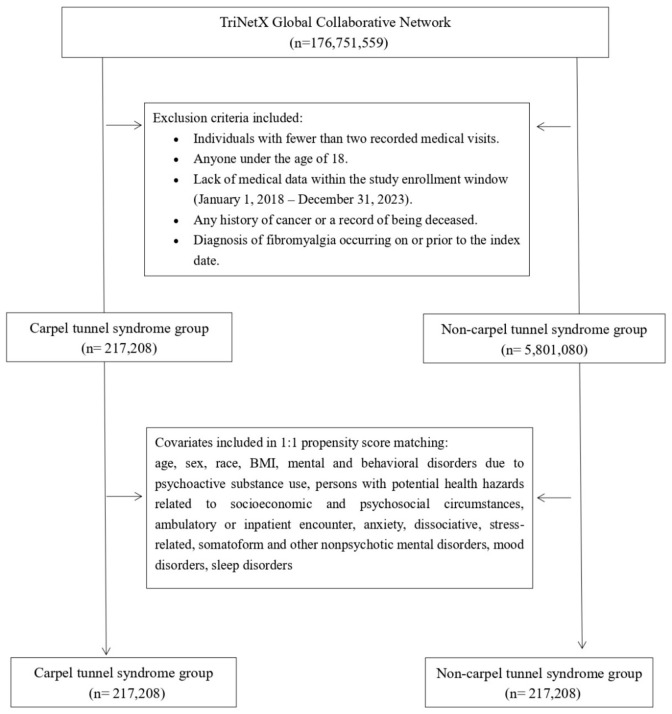
Flowchart of patient selection.

Before matching, the CTS cohort enrolled 217,208 patients, and the CTS-free cohort enrolled 5,801,080 patients (Figure 1). Table 1 shows the SMDs of different characteristics between the CTS and CTS-free cohorts before and after 1:1 propensity score matching. Before matching, CTS patients had a higher rate of BMI greater than 25 kg/m^2^ (58.9% vs. 40.2%; SMD = 0.38) and were older than the control group (mean age, 53.4 ± 15.5 vs. 44.3 ± 18.5 years; SMD = 0.54). CTS patients also presented a higher proportion of ambulatory visits (93.4% vs. 83.8%; SMD = 0.31) and essential hypertension (34.4% vs. 21.0%; SMD = 0.30). Other comorbidities, such as diabetes mellitus (16.6% vs. 7.80%; SMD = 0.27), sleep disorders (17.3% vs. 8.60%; SMD = 0.26), hyperlipidemia (21.2% vs. 13.1%; SMD = 0.22), and mood disorders (18.7% vs. 12.1%; SMD = 0.18), were found more frequently in the CTS cohort. Sex distributions differed modestly, with the CTS cohort having a higher proportion of females (63.8% vs. 55.4%; SMD = 0.17) than males (36.2% vs. 44.4%; SMD = 0.17). These differences became insignificant after propensity score matching.

### 3.2. Cumulative Probability of Fibromyalgia Risk and Sensitivity Analyses

**Figure 2:**
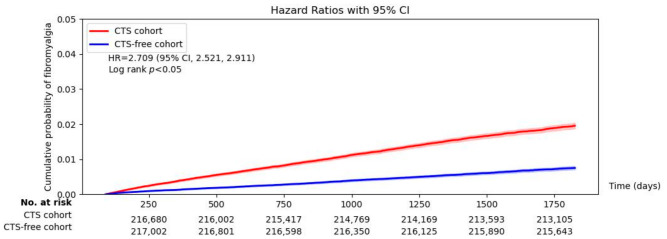
Cumulative probability of fibromyalgia in CTS and CTS-free controls. Legends: CTS, carpal tunnel syndrome; HR, hazard ratio; CI, confidence interval.

As illustrated in Figure 2, fibromyalgia occurred at a higher rate among CTS patients compared with CTS-free controls, with an HR of 2.709 (95% CI: 2.521–2.911) during the up-to-5-year follow-up period. The difference in trends between the two groups was statistically significant, as demonstrated by a log-rank test (*p* < 0.05). The findings of the sensitivity analyses validated those of the main analysis. The crude hazard ratio was significantly elevated in Model 1a (HR = 3.775; 95% CI: 3.623–3.933) and Model 2b, which was matched for age and sex (HR = 3.198; 95% CI: 2.96–3.455). This significantly elevated risk persisted when different washout periods were applied, as shown in Model 1c (HR = 2.854; 95% CI: 2.636–3.09) and Model 2d (HR = 2.76; 95% CI: 2.526–3.016), with washout periods of 12 and 24 months, respectively. Maximum follow-up periods of 1 year and 5 years yielded similar results, as demonstrated in Model 1e (HR = 2.95; 95% CI: 2.56–3.399) and Model 2f (HR = 2.709; 95% CI: 2.521–2.911). When stricter claim-based algorithms were applied, Model 1g (HR = 3.08; 95% CI: 2.656–3.573) and Model 2h (HR = 3.715; 95% CI: 3.402–4.056) demonstrated similarly elevated risks. Finally, when patients with osteoarthritis were used as an active comparator, Model 1i (HR = 1.331; 95% CI: 1.254–1.411) still showed a significantly higher risk of fibromyalgia in CTS patients. When the enrollment window was divided into 2018–2020 and 2021–2023, the association remained significant, indicating that the CTS–fibromyalgia association existed in both the COVID-19 era and the post-COVID-19 era (Table 2).

### 3.3. Stratification Analysis

When stratified by age, CTS patients aged 18–64 years had an HR of 2.820 (95% CI: 2.595–3.065), whereas those aged > 65 years had an HR of 2.717 (95% CI: 2.337–3.159). When stratified by sex, male CTS patients had an HR of 3.018 (95% CI: 2.482–3.672), while female CTS patients had an HR of 2.655 (95% CI: 2.457–2.869) (Table 3).

## 4. Discussion

In this large-scale retrospective cohort study, we found that CTS was associated with a significantly increased risk of fibromyalgia. Individuals with CTS had an approximately 170.9% higher risk of developing fibromyalgia than matched controls (HR 2.709, 95% CI 2.521–2.911). This association remained statistically significant across sensitivity analyses using different model specifications, active-comparator control groups, and stratified analyses by age and sex.

Beyond local tissue alterations, accumulating epidemiological evidence suggests that CTS is associated with a broad range of chronic inflammatory and immune-mediated disorders [17,18,19], indicating that CTS may occur within a more complex biological context than a purely mechanical entrapment neuropathy. Although these observations do not establish a causal role for inflammation in CTS pathogenesis, they suggest that inflammatory and immune-related processes are associated with the disease in at least a subset of patients. Previous evidence also suggests that CTS and fibromyalgia may coexist in clinical settings. Early population-based evidence showed that both conditions were present in 9.7% of clinically examined participants with peripheral joint symptoms, with an estimated odds ratio of 2.4 for having one condition in the presence of the other; however, CTS was diagnosed clinically without nerve conduction studies, and potential selection and information bias were acknowledged [20]. Subsequent electrophysiological evidence found CTS in 20.63% of extremities from patients with fibromyalgia, compared with 2.82% of extremities from controls, suggesting that objective median nerve abnormalities may be more frequent among patients with fibromyalgia [12]. More recent ENMG-based data have been less consistent, showing similar CTS rates between patients with fibromyalgia and controls referred for suspected CTS, with a high normal ENMG rate of 75% [21]. Another recent study using clinical assessment along with electrodiagnostic testing and ultrasonography reported CTS in 35% of patients with fibromyalgia compared with 2.8% of controls, and median nerve ultrasonographic measures were correlated with CTS symptom severity and functional impairment [13]. A large population-based case–control study further showed that patients with fibromyalgia had higher odds of CTS, with an odds ratio of 2.98, and also had higher rates of carpal tunnel release and hand-related healthcare utilization [11]. Current evidence to date supports an increased prevalence of CTS among patients with fibromyalgia; however, the available studies have been limited by small sample sizes and cross-sectional or case–control study designs. Our current study adopted a different analytical strategy than previous studies, which focused on populations of CTS patients, and applied a retrospective cohort design and a large-scale EHR setting to achieve greater evidential strength regarding the association between CTS and fibromyalgia.

The known pathophysiological pathways of both conditions have been partially established. In CTS, intermittent or sustained median nerve compression leads to nerve ischemia and is accompanied by ischemia–reperfusion injury and oxidative stress, resulting in structural and functional changes within both the median nerve and the surrounding subsynovial connective tissue [22,23,24]. In CTS, the subsynovial connective tissue often exhibits fibrotic thickening. This change is frequently associated with increased fibroblast density; larger collagen fibers, especially type III collagen; and enhanced transforming growth factor-beta expression [25]. Increased vascular proliferation has been observed, particularly in diabetic CTS patients [26]. While CTS has traditionally been characterized as noninflammatory fibrosis, recent immunohistochemical studies have demonstrated higher densities of T-lymphocytes in the subsynovial connective tissue compared to controls, though routine histology typically shows minimal to absent inflammatory infiltrate [27]. Histological alterations of the flexor tenosynovium may include fibrotic changes, tissue edema, and vascular hypertrophy; however, normal histological findings are observed in many cases [28]. In contrast, fibromyalgia is widely regarded as a disorder of altered pain processing within the central nervous system. Increased levels of pro-inflammatory cytokines, including TNF-α, IL-6, and IL-8, have been observed in patients with fibromyalgia, suggesting that immune and inflammatory pathways may contribute to symptom generation and persistence [5,29]. Fibromyalgia is also considered a central sensitization syndrome involving dysfunction of pain-processing neurocircuits, resulting in amplified responses to sensory stimuli and enhanced pain perception [30,31]. In addition, previous studies have demonstrated small-fiber abnormalities in a substantial proportion of patients with fibromyalgia, indicating that peripheral nervous system involvement may coexist with central pain dysregulation [32].

Based on these observations, several hypotheses may explain the association between CTS and fibromyalgia. Chronic pain arising from CTS may provide sustained nociceptive input that influences pain perception over time [33,34]. In addition, both conditions have been linked to overlapping biological mechanisms, including neurogenic inflammation and alterations in immune regulation. Shared clinical features, such as obesity, sleep disturbance, psychological distress, and vulnerability to chronic pain, may also partially account for this association. However, these proposed explanations remain speculative in the context of the present study. Because our analysis was based on observational electronic health record data, it cannot determine whether any specific biological pathway underlies the association between CTS and a subsequent diagnosis of fibromyalgia. Therefore, the current findings should not be interpreted as evidence of a causal mechanistic relationship. Rather, the proposed biological explanations should be regarded as hypotheses warranting further investigation in prospective and experimental studies.

This study has several strengths. First, the use of a large-scale database provided a level of data scale that could not be achieved in small-sample studies, allowing for a more robust estimation of the CTS–fibromyalgia association. The large sample size also enhanced statistical power and enabled age- and sex-stratified analyses. Second, propensity score matching across a broad range of covariates enabled more appropriate comparisons between patients with CTS and CTS-free controls.

However, several limitations should be noted. First, this study was based on a retrospective analysis of electronic health record data, which inherently limits causal inference. Accordingly, the results should be interpreted as indicating an association between CTS and fibromyalgia rather than a direct causal relationship. Second, information bias may have contributed to unmeasured or residual confounding because this study relied on electronic health records, which cannot fully capture many clinically relevant characteristics. CTS diagnosis represents a broad clinical spectrum, ranging from mild disease to severe cases requiring surgical intervention. However, detailed indicators of disease severity, including nerve conduction study results, electrophysiological parameters, symptom severity assessments, functional impairment measures, and surgical indications, were not available for analysis. Consequently, we were unable to determine whether the observed association differed according to CTS severity. Likewise, clinical assessments commonly used to characterize fibromyalgia, including symptom-based evaluations and physical examination findings, could not be examined. Patients diagnosed with CTS may also have differed from controls in ways that were not adequately reflected in coded diagnoses. Before the index date, they may have experienced a greater overall burden of pain-related symptoms or psychological distress. Although individuals with a prior fibromyalgia diagnosis were excluded and several comorbid conditions were incorporated into the propensity score matching model, diagnostic codes provide only limited information regarding symptom intensity and overall clinical presentation. As a result, some patients in the CTS cohort may already have had an underlying predisposition to widespread pain or early manifestations of fibromyalgia that had not yet been formally recognized. Such residual confounding is inherent to observational studies using routine clinical data and may partly explain the observed association between CTS and subsequent fibromyalgia diagnosis. Third, medical surveillance bias may have influenced outcome ascertainment. Patients with CTS may undergo repeated clinical evaluations because of persistent pain, functional impairment, or treatment follow-up. Consequently, these individuals may have more opportunities for the recognition and coding of fibromyalgia than individuals in the general population. Although healthcare utilization variables were incorporated into the propensity score matching model, the intensity of medical encounters was not available in the dataset. To further address this concern, we performed an active-comparator analysis using osteoarthritis. Osteoarthritis was selected because it shares several clinical characteristics with CTS, including chronic pain, functional limitation, and regular healthcare contact, all of which may increase opportunities for fibromyalgia recognition. At the same time, it lacks the established systemic inflammatory features and strong epidemiologic association with fibromyalgia observed in conditions such as rheumatoid arthritis. Therefore, osteoarthritis provided a potentially comparable active comparator for evaluating whether the observed association might be explained solely by differential healthcare utilization or chronic musculoskeletal symptoms. However, although CTS remained associated with a higher risk of fibromyalgia compared with osteoarthritis, differential diagnostic intensity may still have contributed to the observed association. Therefore, surveillance bias cannot be completely excluded and should be considered when interpreting the magnitude of the observed hazard ratio. Fourth, misclassification bias related to CTS diagnosis should also be considered. In the TriNetX platform, CTS was identified using ICD-10-CM diagnostic codes, and detailed clinical confirmation was not available. Therefore, we could not determine whether CTS diagnoses were supported by neurophysiological testing, electroneurography, nerve conduction studies, electromyography, or standardized neurological examinations, such as provocative maneuvers and sensory or motor assessments. The absence of these data limited our ability to verify objective compression-related changes in the sensory or motor fibers of the median nerve. To improve diagnostic specificity, we conducted sensitivity analyses using stricter claim-based algorithms, including CTS diagnosis with median nerve release surgery and CTS diagnosis with a prescription for nonsteroidal anti-inflammatory drugs. The association with subsequent coded fibromyalgia diagnosis remained significant in these models. However, these approaches cannot fully replace direct neurophysiological confirmation, and some degree of CTS misclassification may have persisted.

## 5. Conclusions

In conclusion, we report that CTS was associated with a subsequent coded diagnosis of fibromyalgia. The association was observed across age- and sex-stratified analyses and several sensitivity analyses, including an active-comparator analysis using osteoarthritis. However, these findings should not be interpreted as evidence that CTS causes fibromyalgia or directly increases biological susceptibility to fibromyalgia, given the inherent limitations of EHR-based research and the retrospective cohort design. Rather, CTS may represent a clinical marker associated with a later diagnosis of fibromyalgia, supporting the need for greater awareness of broader pain-related symptoms in patients with CTS. Further prospective and mechanistic studies are warranted to clarify the temporal relationship and possible shared pathophysiological features between these conditions.

## Figures and Tables

**Table 1:** Baseline characteristics.

	Before Matching			After Matching ^a^		
	CTS Cohort (*n* = 217,208)	Non-CTS Control Cohort (*n* = 5,801,080)	SMD	CTS Cohort (*n* = 217,208)	Non-CTS Control Cohort (*n* = 217,208)	SMD
Age at index						
Mean ± SD	53.4 ± 15.5	44.3 ± 18.5	0.54	53.4 ± 15.5	53.4 ± 15.5	0.00
Sex						
Female	138,557 (63.8)	3,215,273 (55.4)	0.17	138,557 (63.8)	138,383 (63.7)	0.00
Male	78,566 (36.2)	2,577,891 (44.4)	0.17	78,566 (36.2)	78,715 (36.2)	0.00
Unknown gender	85 (<0.1)	7916 (0.1)	0.03	85 (<0.1)	110 (0.1)	0.01
Race, *n* (%)						
White	129,784 (59.8)	3,548,802 (61.2)	0.03	129,784 (59.8)	129,904 (59.8)	0.00
Black or African American	31,122 (14.3)	775,032 (13.4)	0.03	31,122 (14.3)	34,823 (16.0)	0.05
Asian	10,547 (4.9)	250,809 (4.3)	0.03	10,547 (4.9)	9488 (4.4)	0.02
American Indian or Alaska Native	1264 (0.6)	20,021 (0.3)	0.03	1264 (0.6)	879 (0.4)	0.03
Native Hawaiian or Other Pacific Islander	1170 (0.5)	17,271 (0.3)	0.04	1170 (0.5)	732 (0.3)	0.03
Other race	7325 (3.4)	310,879 (5.4)	0.10	7325 (3.4)	10,609 (4.9)	0.08
Unknown race	35,996 (16.6)	878,266 (15.1)	0.04	35,996 (16.6)	30,773 (14.2)	0.07
BMI, *n* (%)						
≥25 (kg/m^2^)	128,023 (58.9)	2,334,652 (40.2)	0.38	128,023 (58.9)	128,196 (59.0)	0.00
Medical utilization status, *n* (%)						
Visit: ambulatory	202,834 (93.4)	4,859,461 (83.8)	0.31	202,834 (93.4)	202,994 (93.5)	0.00
Visit: inpatient encounter	59,667 (27.5)	1,042,659 (18.0)	0.23	59,667 (27.5)	59,771 (27.5)	0.00
Socioeconomic status, *n* (%)						
Persons with potential health hazards related to socioeconomic and psychosocial circumstances	6117 (2.8)	133,671 (2.3)	0.03	6117 (2.8)	5871 (2.7)	0.01
Lifestyle, *n* (%)						
Mental and behavioral disorders due to psychoactive substance use	34,344 (15.8)	526,889 (9.1)	0.20	34,344 (15.8)	34,239 (15.8)	0.00
Comorbidities, *n* (%)						
Essential hypertension	74,646 (34.4)	1,217,268 (21.0)	0.30	74,646 (34.4)	74,531 (34.3)	0.00
Anxiety, dissociative, stress-related, somatoform and other nonpsychotic mental disorders	48,103 (22.1)	956812 (16.5)	0.14	48,103 (22.1)	48,068 (22.1)	0.00
Hyperlipidemia	46,041 (21.2)	758,440 (13.1)	0.22	46,041 (21.2)	46,517 (21.4)	0.01
Mood disorders	40,606 (18.7)	703,595 (12.1)	0.18	40,606 (18.7)	40,521 (18.7)	0.00
Sleep disorders	37,660 (17.3)	498,765 (8.6)	0.26	37,660 (17.3)	37,495 (17.3)	0.00
Diabetes mellitus	35,994 (16.6)	451,711 (7.8)	0.27	35,994 (16.6)	28,973 (13.3)	0.09
Vitamin D deficiency	25,539 (11.8)	480,976 (8.3)	0.12	25,539 (11.8)	27,263 (12.6)	0.02
Chronic ischemic heart disease	15,109 (7.0)	202,389 (3.5)	0.16	15,109 (7.0)	13,790 (6.3)	0.02
Chronic kidney disease	9040 (4.2)	136,356 (2.4)	0.10	9040 (4.2)	9227 (4.2)	0.00
Psoriasis	3066 (1.4)	50,009 (0.9)	0.05	3066 (1.4)	2651 (1.2)	0.02
Rheumatoid arthritis	1822 (0.8)	13,454 (0.2)	0.08	1822 (0.8)	859 (0.4)	0.06
Systemic lupus erythematosus	1125 (0.5)	12,583 (0.2)	0.05	1125 (0.5)	803 (0.4)	0.02
Ulcerative colitis	790 (0.4)	19,394 (0.3)	0.00	790 (0.4)	941 (0.4)	0.01
Crohn’s disease	745 (0.3)	19,805 (0.3)	0.00	745 (0.3)	917 (0.4)	0.01
Ankylosing spondylitis	558 (0.3)	4699 (0.1)	0.04	558 (0.3)	239 (0.1)	0.03

CTS: carpal tunnel syndrome; SMD, standardized mean difference (when >0.1 represents significant difference between groups). ^a^ The covariates matched included age; sex; race; BMI; mental and behavioral disorders due to psychoactive substance use; persons with potential health hazards related to socioeconomic and psychosocial circumstances; ambulatory or inpatient encounter; and anxiety, dissociative, stress-related, somatoform and other nonpsychotic mental disorders; mood disorders; and sleep disorders.

**Table 2:** Hazard ratio of fibromyalgia under various models.

Various Matching Covariates	Model 1 ^a^	Model 2 ^b^
Non-CTS controls	1.00	1.00
CTS patients	3.775 (3.623, 3.933)	3.198 (2.96, 3.455)
Various washout periods	Model 1 ^c^	Model 2 ^d^
Non-CTS controls	1.00	1.00
CTS patients	2.854 (2.636, 3.09)	2.76 (2.526, 3.016)
Various maximum follow-up times	Model 1 ^e^	Model 2 ^f^
Non-CTS controls	1.00	1.00
CTS patients	2.95 (2.56, 3.399)	2.709 (2.521, 2.911)
Various claim-based algorithms	Model 1 ^g^	Model 2 ^h^
Non-CTS controls	1.00	1.00
CTS patients	3.08 (2.656, 3.573)	3.715 (3.402, 4.056)
Various active comparator	Model 1 ^i^	
Non-CTS controls	1.00	
CTS patients	1.331 (1.254, 1.411)	
Various enrollment window	Model 1 ^j^	Model 2 ^k^
Non-CTS controls	1.00	1.00
CTS patients	2.78 (2.53, 3.055)	2.719 (2.48, 2.982)

CTS, carpal tunnel syndrome; aside from analyses applying various matching covariates, all other analyses applied 1:1 propensity score matching with covariates including age; sex; race; BMI; mental and behavioral disorders due to psychoactive substance use; persons with potential health hazards related to socioeconomic and psychosocial circumstances; ambulatory or inpatient encounter; and anxiety, dissociative, stress-related, somatoform and other nonpsychotic mental disorders; mood disorders; and sleep disorders. If not specified, the washout period was set as 3 months after index date, and the follow-up period was up to 5 years. ^a^ In this model, results were compared before propensity score matching was performed. ^b^ In this model, propensity score matching was performed based on the covariates of age and sex. ^c^ A 12-month washout period was applied in this model; cases of incident fibromyalgia occurring during this period were excluded from subsequent analyses. ^d^ A 24-month washout period was applied in this model; cases of incident fibromyalgia occurring during this period were excluded from subsequent analyses. ^e^ Follow-up was conducted for up to one year. ^f^ Follow-up was conducted for up to five years. ^g^ Patients were included in the CTS cohort if they had a coded diagnosis of carpal tunnel syndrome (G56.0) and had undergone median nerve release surgery (procedure code 64721). ^h^ Patients were included in the CTS cohort if they had a coded diagnosis of carpal tunnel syndrome (G56.0) and were prescribed non-steroidal anti-inflammatory analgesics (VA code CN104). ^i^ Patients with osteoarthritis were used as the comparison group. ^j^ The enrollment window was set between 1 January 2018 and 31 December 2020 ^k^ The enrollment window was set between 1 January 2021 and 31 December 2023.

**Table 3:** Stratification analysis of fibromyalgia risk in CTS patients in 5-year follow-up.

	Cases of New-Onset Fibromyalgia		
Subgroups	CTS Cohort No. of Outcome Events (%)	Control Cohort No. of Outcome Events (%)	Hazard Ratio (95% CI) ^a^
Age at index date			
18–64 years old	1994 (1.4)	769 (0.5)	2.820 (2.595, 3.065)
≥65 years old	590 (0.8)	238 (0.3)	2.717 (2.337, 3.159)
Sex			
Male	373 (0.5)	137 (0.2)	3.018 (2.482, 3.672)
Female	2210 (1.6)	903 (0.7)	2.655 (2.457, 2.869)

CTS, carpal tunnel syndrome; CI, confidence interval. ^a^ The covariates matched included age; sex; race; BMI; mental and behavioral disorders due to psychoactive substance use; persons with potential health hazards related to socioeconomic and psychosocial circumstances; ambulatory or inpatient encounter; and anxiety, dissociative, stress-related, somatoform and other nonpsychotic mental disorders; mood disorders; and sleep disorders.

## Data Availability

Data in this study were retrieved from TriNetX Research Network. All data available in the database were administrated by the TriNetX platform. Detailed information can be retrieved at the official website of the research network (https://trinetx.com, accessed on 1 June 2026).

## Supplementary Materials

The following supporting information can be downloaded at: https://www.mdpi.com/article/10.3390/life16071059/s1, Table S1: Utilized ICD-10-CM codes.
