# Supplementary material for: Carpal Tunnel Syndrome Is Associated with Increased Risk of Fibromyalgia: A Retrospective Cohort Study
Source: Life (Basel). 2026 Jun 25;16(7):1059. doi: 10.3390/life16071059 (PMC13412866; doi:10.3390/life16071059)
Supplement: Supplementary file 1 [file life-16-01059-s001.zip › life-4364819-supplementary.pdf]

Supplementary Files

Supplementary Tables

Table S1. Utilized ICD-10-CM codes [a]

**Description**	**ICD-10-CM codes**
Study population	
Carpel tunnel syndrome	ICD-10-CM: G56.0
Outcome event	
Fibromyalgia	ICD-10-CM: M79.7
Other diseases or covariates	
Essential hypertension	ICD-10-CM: I10
Diabetes mellitus	ICD-10-CM: E08-E13
Hyperlipidemia	ICD-10-CM: E78.5
Chronic ischemic heart disease	ICD-10-CM: I25
Vitamin D deficiency	ICD-10-CM: E55
Chronic kidney disease	ICD-10-CM: K18
Rheumatoid arthritis	ICD-10-CM: M05
Ankylosing spondylitis	ICD-10-CM: M45
Crohn's disease	ICD-10-CM: K50
Ulcerative colitis	ICD-10-CM: K51
Psoriasis	ICD-10-CM: L40
Systemic lupus erythematosus	ICD-10-CM: M32
Anxiety, dissociative, stress-related, somatoform and other nonpsychotic mental	ICD-10-CM: F40-F48

disorders	
Mood disorders	ICD-10-CM: F30-F39
Sleep disorders	ICD-10-CM: G47
Persons with potential health hazards related to socioeconomic and psychosocial circumstances	ICD-10-CM: Z55-Z65
Mental and behavioral disorders due to psychoactive substance use	ICD-10-CM: F10-F19
Encounter for adult general examination	ICD-10-CM: Z00.0

[a]ICD-10-CM: International Classification of Diseases, Tenth Revision, Clinical Modification
